# Supplementary material for: Mitochondria regulate intracellular coenzyme Q transport and ferroptotic resistance via STARD7
Source: Nat Cell Biol. 2023 Jan 19;25(2):246–57. doi: 10.1038/s41556-022-01071-y (PMC9928583; doi:10.1038/s41556-022-01071-y)
Supplement: Supplementary file 1 — Reporting Summary [file 41556_2022_1071_MOESM1_ESM.pdf]

nature portfolio

Corresponding author(s): Thomas Langer

Last updated by author(s): Nov 14, 2022

Reporting Summary

Nature Portfolio wishes to improve the reproducibility of the work that we publish. This form provides structure for consistency and transparency in reporting. For further information on Nature Portfolio policies, see our Editorial Policies and the Editorial Policy Checklist.

Statistics

For all statistical analyses, confirm that the following items are present in the figure legend, table legend, main text, or Methods section.

n/a	Confirmed	
☐	☒	The exact sample size (*n*) for each experimental group/condition, given as a discrete number and unit of measurement
☐	☒	A statement on whether measurements were taken from distinct samples or whether the same sample was measured repeatedly
☐	☒	The statistical test(s) used AND whether they are one- or two-sided<br>*Only common tests should be described solely by name; describe more complex techniques in the Methods section.*
☒	☐	A description of all covariates tested
☒	☐	A description of any assumptions or corrections, such as tests of normality and adjustment for multiple comparisons
☐	☒	A full description of the statistical parameters including central tendency (e.g. means) or other basic estimates (e.g. regression coefficient) AND variation (e.g. standard deviation) or associated estimates of uncertainty (e.g. confidence intervals)
☒	☐	For null hypothesis testing, the test statistic (e.g. *F*, *t*, *r*) with confidence intervals, effect sizes, degrees of freedom and *P* value noted<br>*Give P values as exact values whenever suitable.*
☒	☐	For Bayesian analysis, information on the choice of priors and Markov chain Monte Carlo settings
☒	☐	For hierarchical and complex designs, identification of the appropriate level for tests and full reporting of outcomes
☐	☒	Estimates of effect sizes (e.g. Cohen's *d*, Pearson's *r*), indicating how they were calculated

Our web collection on statistics for biologists contains articles on many of the points above.

Software and code

Policy information about availability of computer code

Data collection	No software was used
Data analysis	Data was analyzed using Instant clue software 0.11/0.53. Raw data (Mass spec): Spectraraut 15.7.220308.50606 (Fractionation data). DIA.NN 1.8 (tissue samples). https://github.com/hnolCol/instantclue. For metabolomics data (Lipids) Tracefinder 4.1/5.1 from Thermo Fischer was used. Incucyte Base Analysis Software (Included with Incucyte® Live-Cell Analysis System) was used for cell death analysis. Seahorse data was analyzed by Seahorse Wave Desktop Software 2.6.

For manuscripts utilizing custom algorithms or software that are central to the research but not yet described in published literature, software must be made available to editors and reviewers. We strongly encourage code deposition in a community repository (e.g. GitHub). See the Nature Portfolio guidelines for submitting code & software for further information.

Data

Policy information about availability of data

All manuscripts must include a data availability statement. This statement should provide the following information, where applicable:

- Accession codes, unique identifiers, or web links for publicly available datasets
- A description of any restrictions on data availability
- For clinical datasets or third party data, please ensure that the statement adheres to our policy

Mass spectrometry data have been deposited in ProteomeXchange with the primary accession code PXD033064. Previously published gene expression data that were re-analyzed here are available through the Cancer Thera-peutics Response Portal (http://www.broadinstitute.org/ctrp) [3]. Numerical source data and unprocessed blots have been provided in Source Data. All other data supporting the findings of this study are available from the corresponding author on

reasonable request.

Field-specific reporting

Please select the one below that is the best fit for your research. If you are not sure, read the appropriate sections before making your selection.

☒ Life sciences ☐ Behavioural & social sciences ☐ Ecological, evolutionary & environmental sciences

For a reference copy of the document with all sections, see nature.com/documents/nr-reporting-summary-flat.pdf

Life sciences study design

All studies must disclose on these points even when the disclosure is negative.

Sample size	No sample size was calculated. No statistical test was performed to determine the sample size. The sample size included at lease 3 biological replicates where statistical test was performed.
Data exclusions	No data was excluded
Replication	All n are shown separately in each figure. All attempts at replication were successful.
Randomization	Proteomics data and metabolomics data were randomised. For cell culture experiments, samples were allocated in different groups either based on genotypes or based on different treatments at different time-points. Whenever possible different experimental groups were handled together. While doing incucyte experiments, control/wildtype were measured first and then the treated/knockout samples. Covariates do not apply to cell culture experiments, since all the experiments were performed in the same cell culture room, all the cells were cultured in the same DMEM medium and put in the same incubators.
Blinding	Proteomics and metabolomics measurements were blinded. Samples were not blinded for tissue culture experiments. Since most of the experiments were done by the first author and samples were labeled in the tissue culture for the treatments/time-points, it was hard to not know the groups and therefore, the experimenter was always aware of the experimental groups.

Reporting for specific materials, systems and methods

We require information from authors about some types of materials, experimental systems and methods used in many studies. Here, indicate whether each material, system or method listed is relevant to your study. If you are not sure if a list item applies to your research, read the appropriate section before selecting a response.

Materials & experimental systems

n/a	Involved in the study	
☐	☒	Antibodies
☐	☒	Eukaryotic cell lines
☒	☐	Palaeontology and archaeology
☐	☒	Animals and other organisms
☒	☐	Human research participants
☒	☐	Clinical data
☒	☐	Dual use research of concern

Methods

n/a	Involved in the study	
☒	☐	ChIP-seq
☒	☐	Flow cytometry
☒	☐	MRI-based neuroimaging

Antibodies

Antibodies used	SMAC (MBL, JM-3298-100) dilution 1:1000, STARD7 (Proteintech 15689-1-AP) dilution 1:2500, FLAG (WAKO 018-22381) dilution 1:1000, SDHA (Abcam Ab14715) dilution 1:10000, TRANSFERRIN (Invitrogen 13-6800) dilution 1:1000, CLPP (Sigma HPA010649) dilution 1:1000, YME1L (Proteintech 11510-1-AP) dilution 1:1000, MIC60 (Nobus Biologicals 100-1919) dilution 1:1000, VDAC2 (Proteintech 11663-1-AP) dilution 1:1000. PARL antibody is defined previously [1], dilution 1:1000
Validation	Antibodies STARD7 (Extended data fig. 1e) and PARL (Extendend data fig. 1c) are validated by observing no bands in the knockout cell lines of these proteins. FLAG antibody was validated by using overexpressed Flag-tagged proteins (Extended data fig. 1c). SMAC and SDHA antibody has been validated before in our lab (Saita et al., 2017 Nature Cell Biology). YME1l has been validated before in our lab (MacVicar et al., 2019 Nature). Transferrin, CLPP, antibody has been verified by the manufacturer using knockdown experiments. MIC60 validated in PMID: 34037656 according to manufacturer. VDAC2 vaildated by manufacturer using immunoprecipitation here https://www.ptglab.com/products/VDAC2-Antibody-11663-1-AP.htm.

Eukaryotic cell lines

Policy information about cell lines

Cell line source(s)	HCT116: ATCC, catalog number: CCL-247. HeLa (CCL-2) cells were purchased from ATCC. HEK293T cells were described in PMID: 31695197 but only used for virus production and no biological interpretations were based on HEK293T cells. Further information regarding generation of knockout cell line details are included in the Methods section.
Authentication	Cell lines were not authenticated
Mycoplasma contamination	All the cell lines were routinely tested for mycoplasma and only when tested negative were used for the experiments.
Commonly misidentified lines (See ICLAC register)	No commonly misidentified lines were used in this study

Animals and other organisms

Policy information about studies involving animals; ARRIVE guidelines recommended for reporting animal research

Laboratory animals	C57BL/6N mice, male 5-weeks old mice were used. Mice were maintained at the specific-pathogen-free animal facility of the Cologne Excellence Cluster on Cellular Stress Responses in Aging-Associated Diseases (CECAD) Research Centre with 12 h light cycle and regular chow diet.
Wild animals	No Wildtype animals were used in the study.
Field-collected samples	No field collected samples were used in this study.
Ethics oversight	All animal experiments were approved by Landesamt für Natur, Umwelt und Verbraucherschutz Nordrhein-Westfalen, Germany and the Cologne Excellence Cluster on Cellular Stress Responses in Aging-Associated Diseases (CECAD) mouse facility regulations.

Note that full information on the approval of the study protocol must also be provided in the manuscript.
