# Supplementary figures and images for: Mitochondria regulate intracellular coenzyme Q transport and ferroptotic resistance via STARD7
Source: Nat Cell Biol. 2023 Jan 19;25(2):246–57. doi: 10.1038/s41556-022-01071-y (PMC9928583; doi:10.1038/s41556-022-01071-y)

Extended Data Fig. 1

c

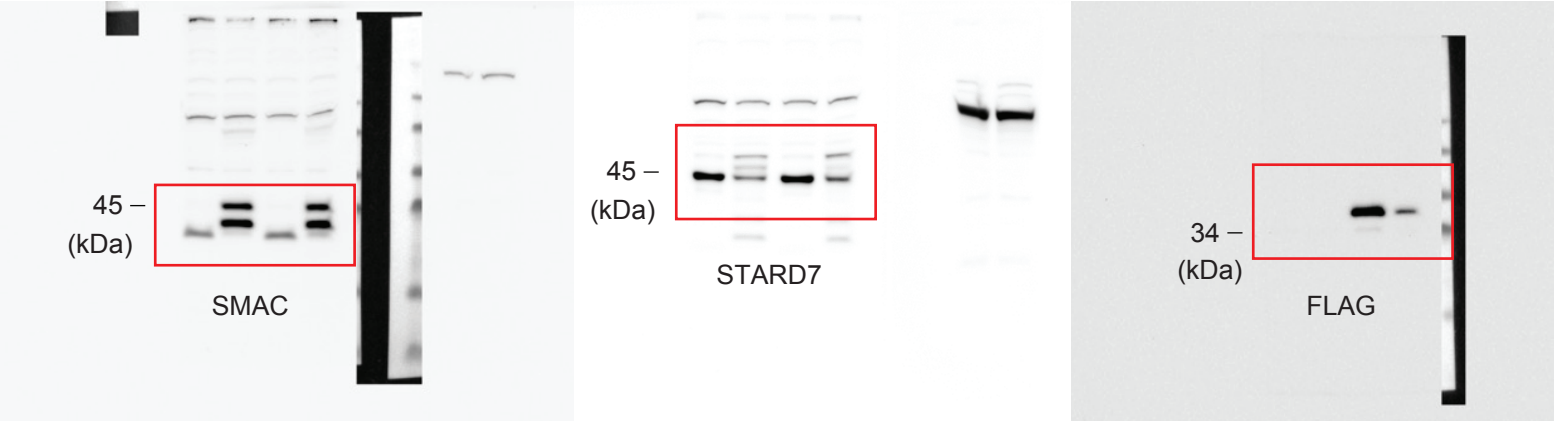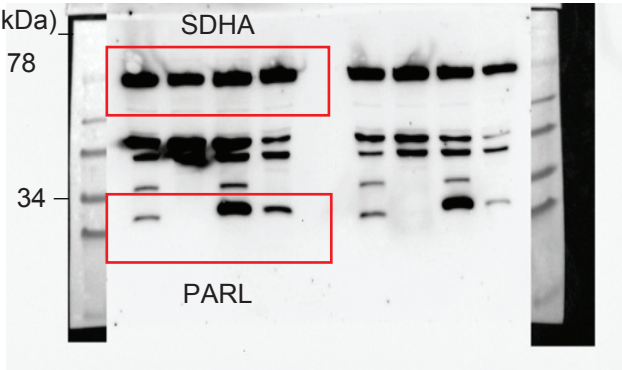

e

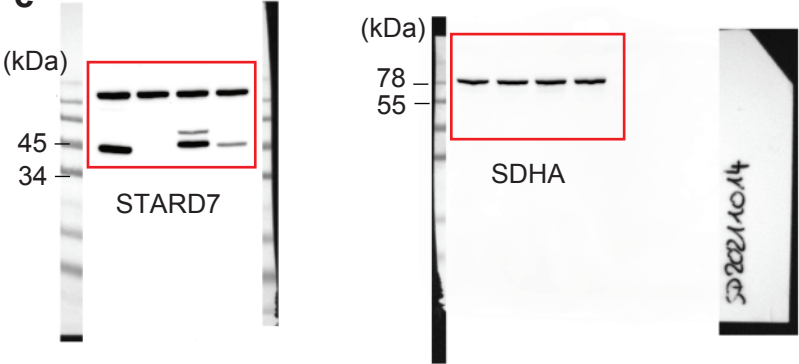

h

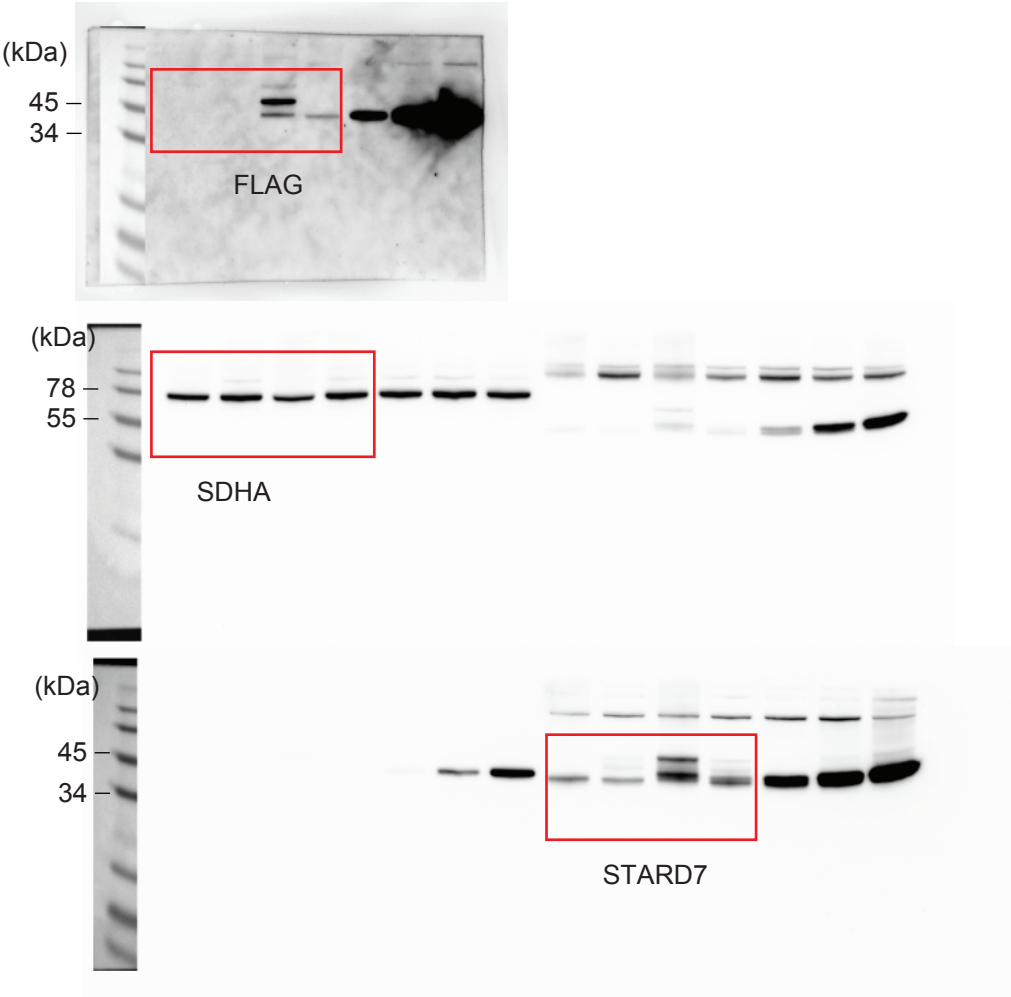

Supplement: Source Data Extended Data Fig./Table 1 — Unprocessed western blots. [file 41556_2022_1071_MOESM11_ESM.pdf]

Extended Data Fig. 2

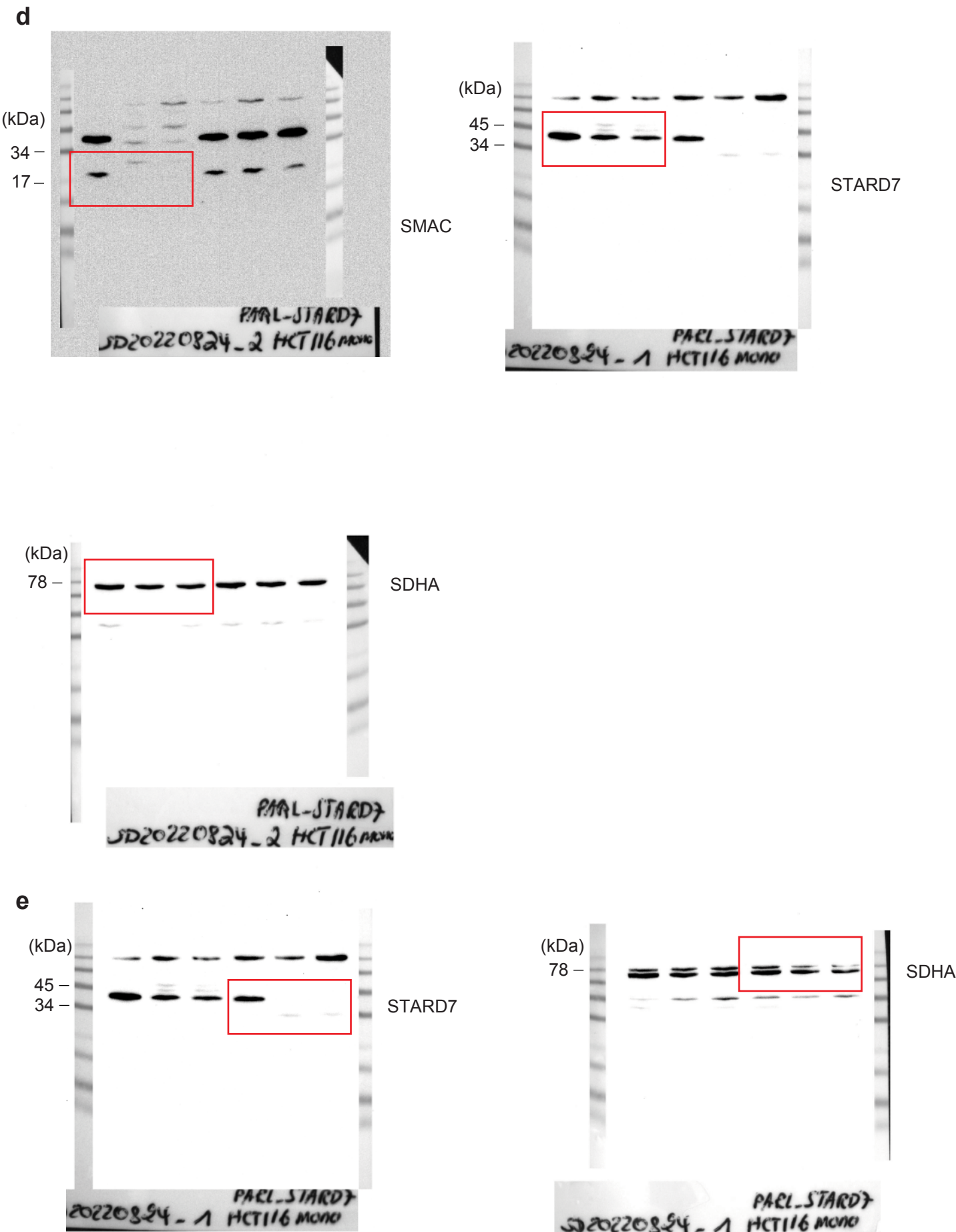

Supplement: Source Data Extended Data Fig./Table 2 — Unprocessed western blots. [file 41556_2022_1071_MOESM13_ESM.pdf]

Extended Data Fig. 3

**a**

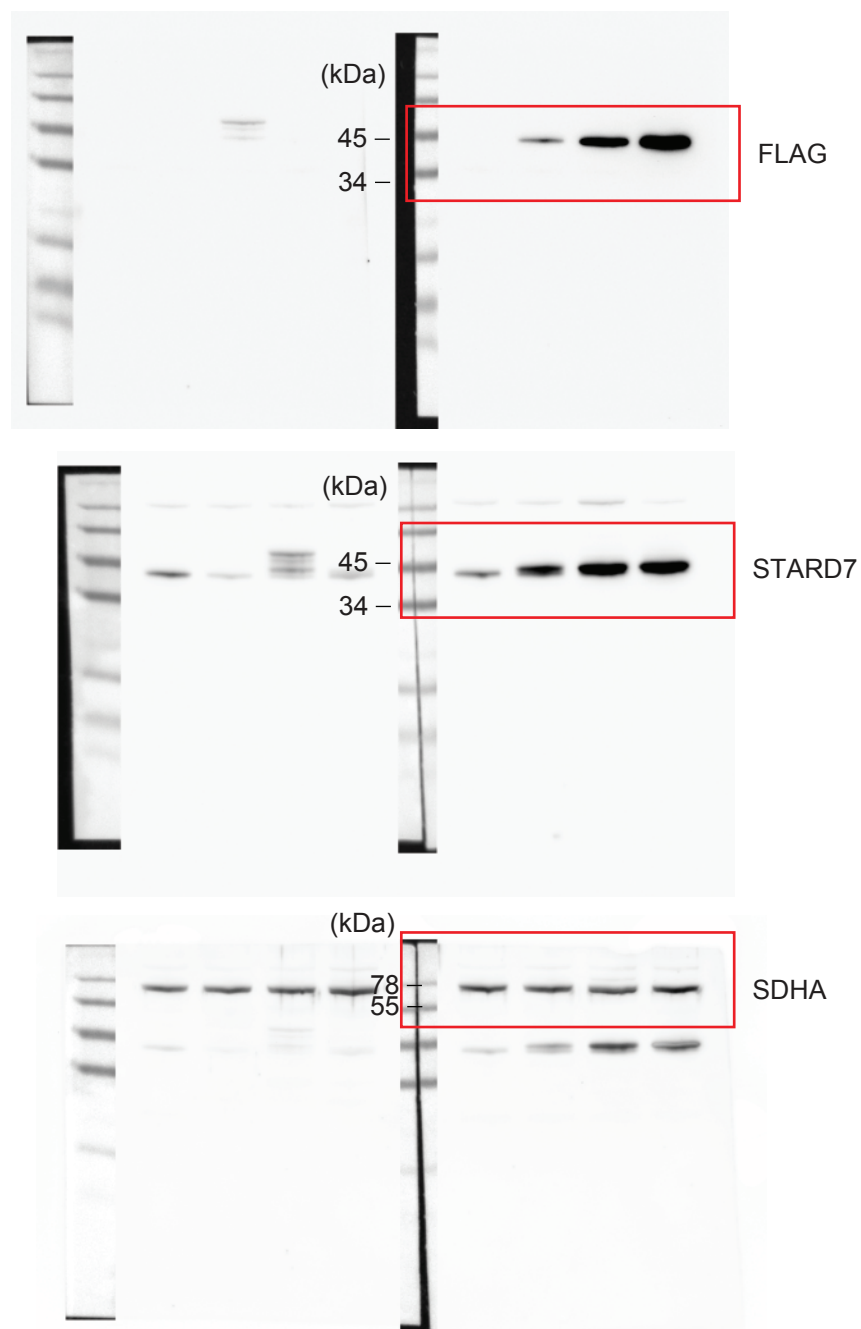

Supplement: Source Data Extended Data Fig./Table 3 — Unprocessed western blots. [file 41556_2022_1071_MOESM15_ESM.pdf]
