# Supplementary material for: Mitochondria regulate intracellular coenzyme Q transport and ferroptotic resistance via STARD7
Source: Nat Cell Biol. 2023 Jan 19;25(2):246–57. doi: 10.1038/s41556-022-01071-y (PMC9928583; doi:10.1038/s41556-022-01071-y)
Supplement: Source Data Extended Data Fig./Table 4 — Unprocessed western blots. [file 41556_2022_1071_MOESM17_ESM.pdf]

Extended Data Fig. 4

a

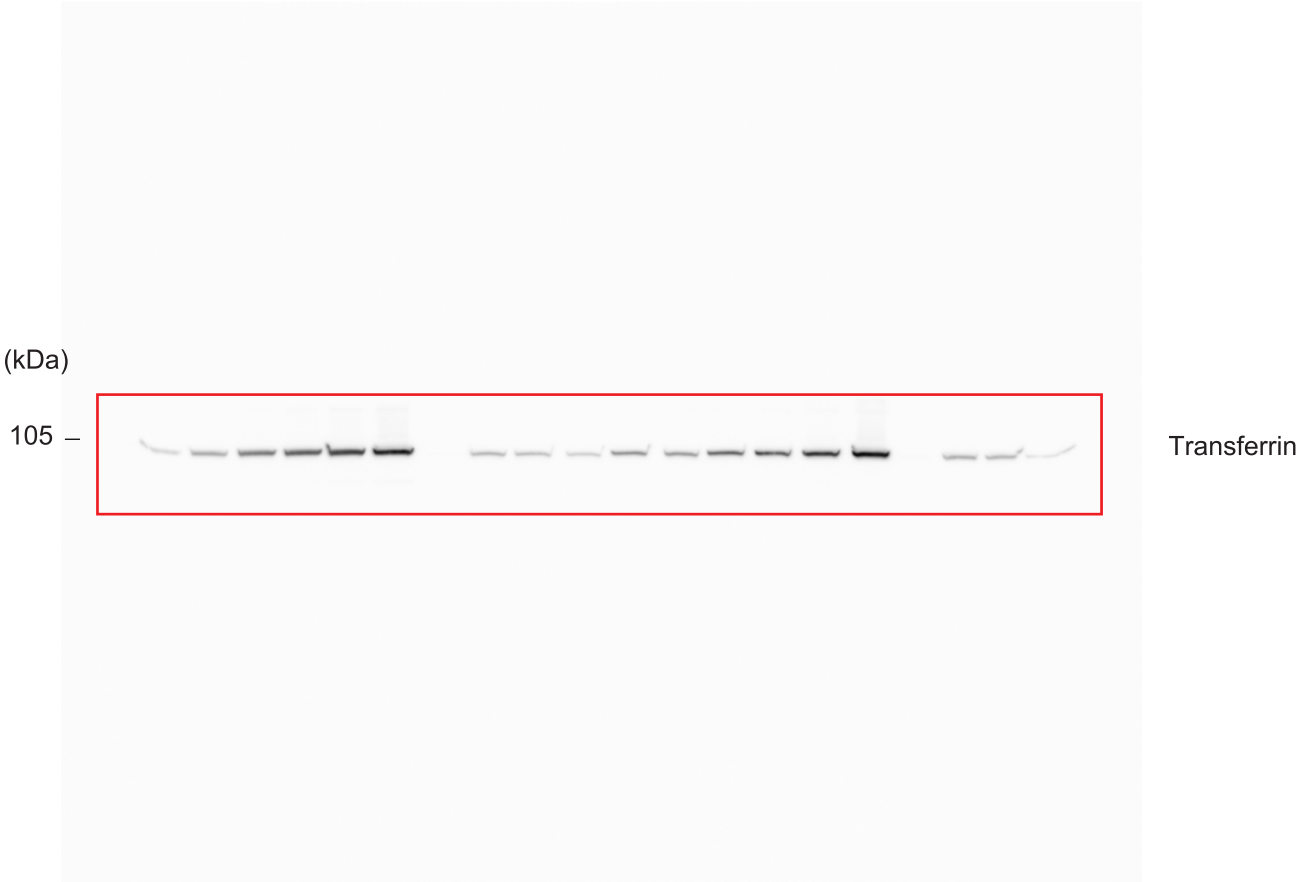

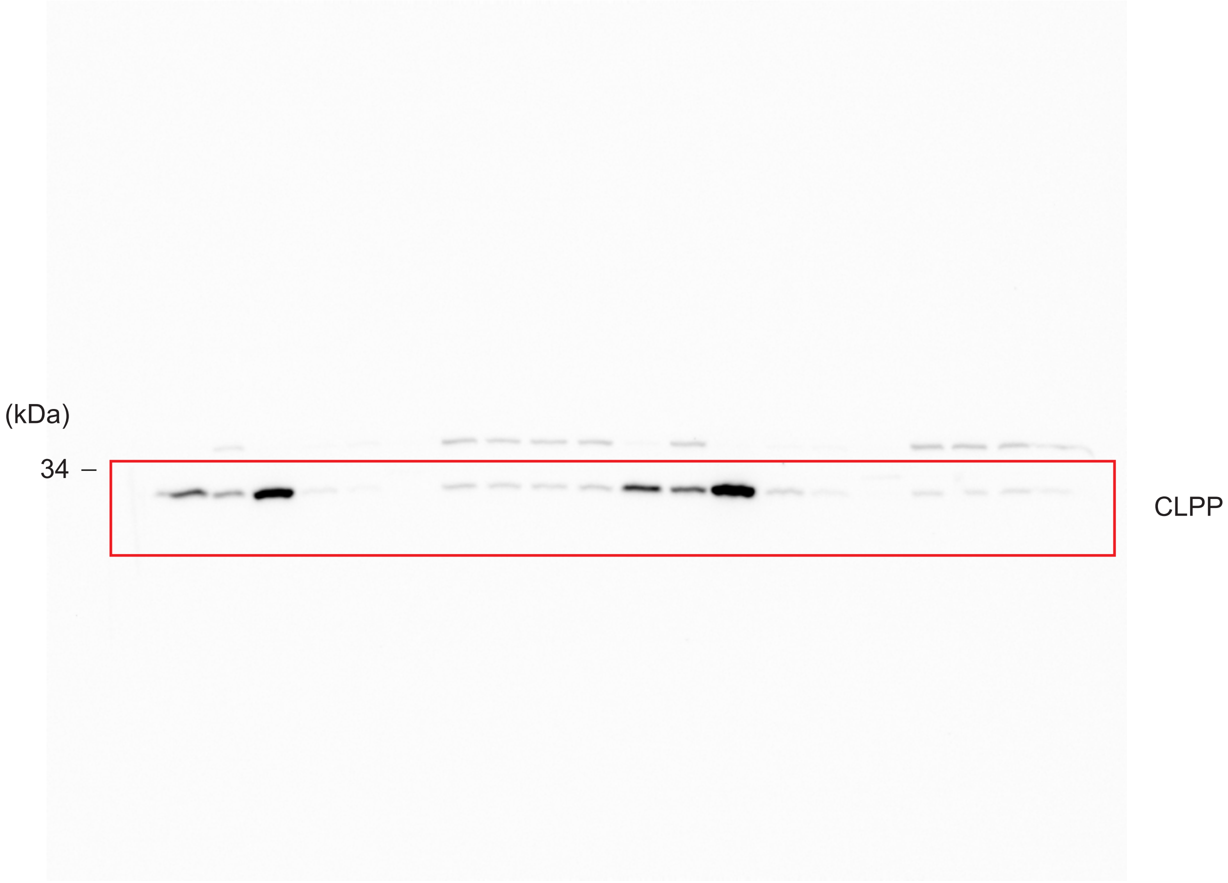

Extended Data Fig. 4

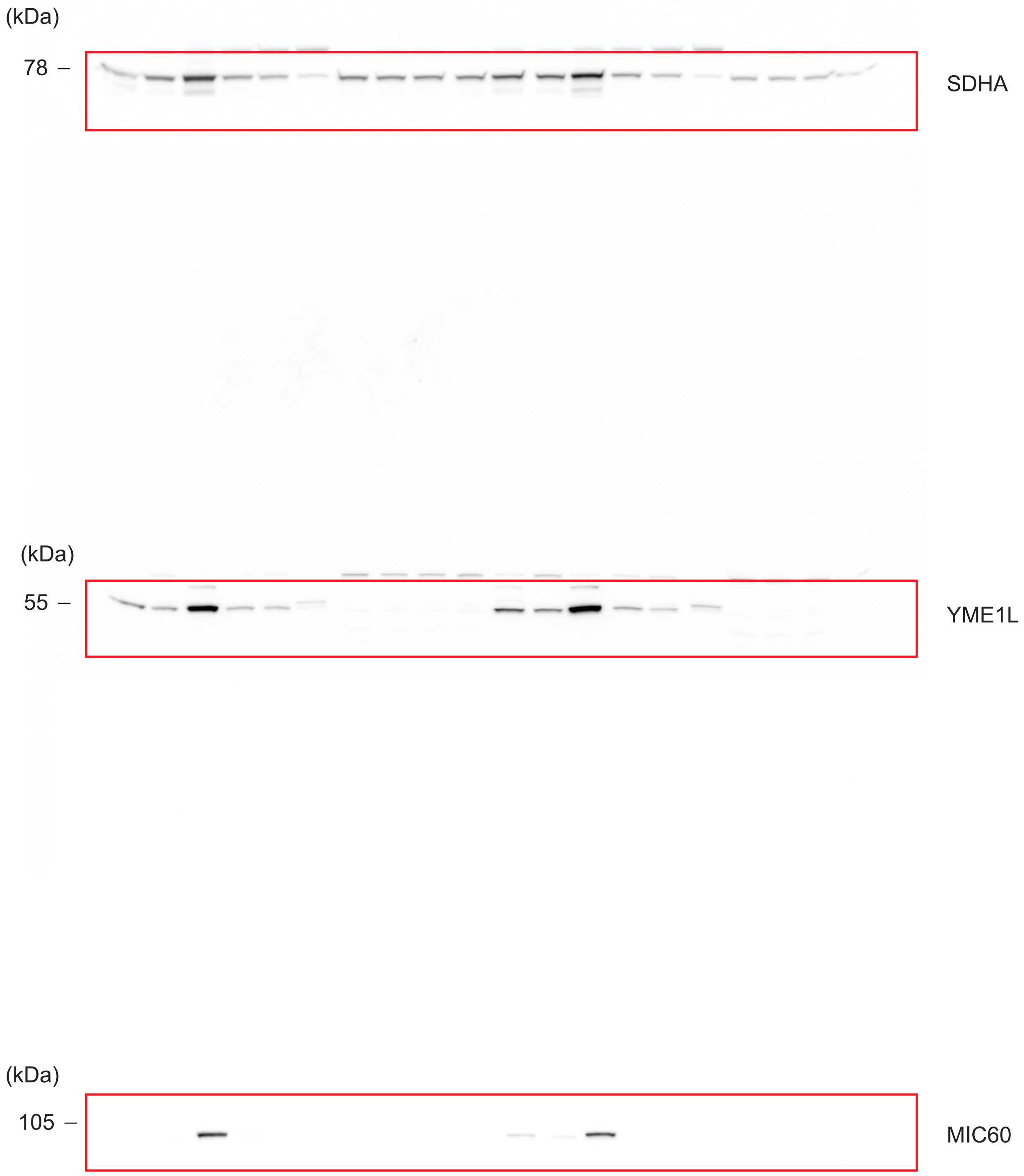

Extended Data Fig. 4

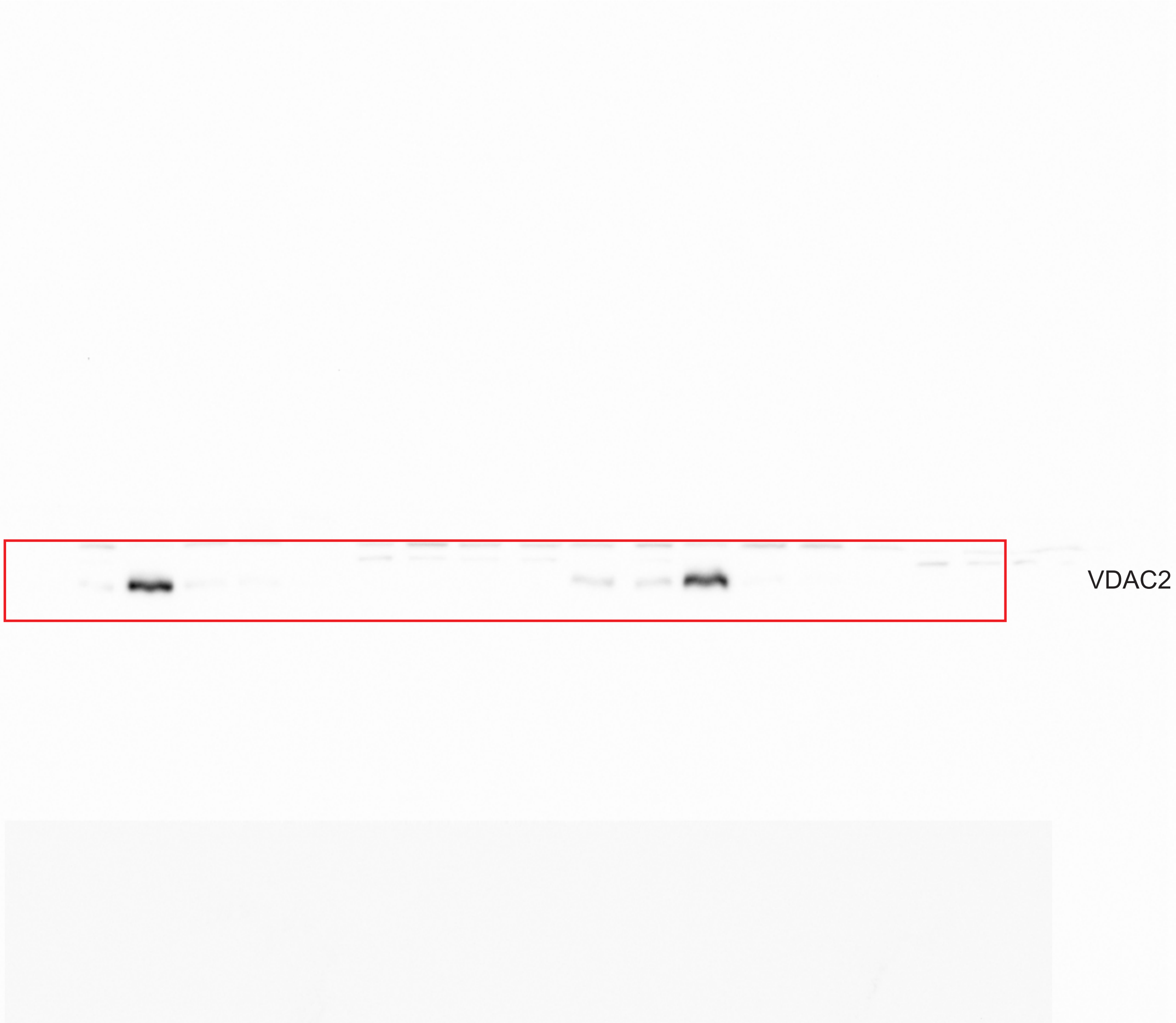

(kDa)

34 –

VDAC2

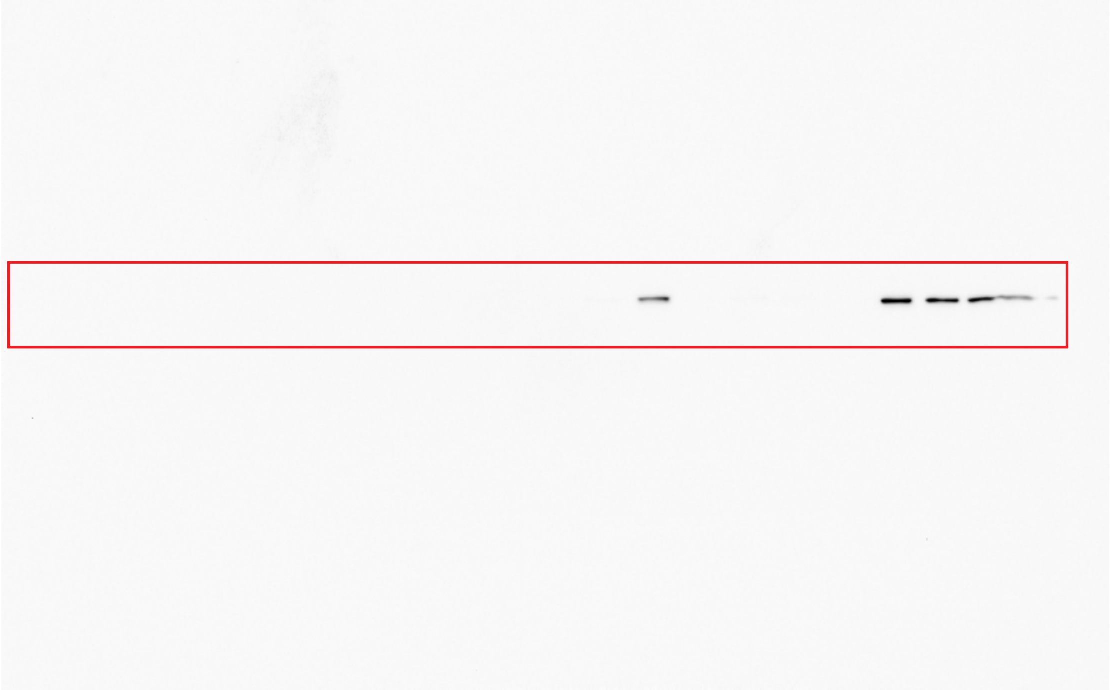

(kDa)

45 –

FLAG
